# Determinants of depression among nursing students in Cameroon: a cross-sectional analysis

**DOI:** 10.1186/s12912-020-00424-y

**Published:** 2020-04-17

**Authors:** Tsi Njim, Clarence Mbanga, Dave Mouemba, Haman Makebe, Louise Toukam, Belmond Kika, Isabelle Mulango

**Affiliations:** 1 Health and Human Development (2HD) Research Group, Douala, Littoral region Cameroon; 2 Mankon Sub-divisional Hospital, Bamenda, North west region Cameroon; 3 Regional Hospital Annex Kousseri, Garoua, Far north region Cameroon; 4 Faculty of Health Sciences, University of Buea, Buea, South west region Cameroon; 5 Faculty of Health Sciences, University of Bamenda, Bamenda, North west region, Cameroon; 6 District Hospital Ekondo-Titi, Ekondo-Titi, South west region Cameroon; 7 District Hospital Kumba, Kumba, South west region Cameroon

**Keywords:** Burnout syndrome, Nursing students, Cameroon, Oldenburg burnout inventory, Patient health Questionnaire-9

## Abstract

**Background:**

Nursing students are highly susceptible to depression given the heavy workload and clinical demands of the curriculum. Depression has negative impacts on their health and academic performance. This study aimed to determine the determinants of depression amongst nursing students in the English-speaking regions of Cameroon.

**Methods:**

A cross-sectional analysis of 447 nursing students recruited from a combination of state-owned and private nursing institutions in the English-speaking regions of Cameroon from January – April 2018 was carried out. Independent correlates of depression were determined using multivariable regression analysis, with the level of significance set at 95%.

**Results:**

The overall prevalence of depression (Patient Health Questionnaire - 9 > 4) and major depressive disorder (Patient Health Questionnaire - 9 > 9) in these students was 69.57 and 26.40% respectively. Independent correlates of depression were found to be: total Oldenburg Burnout Inventory score (aOR: 1.18; 95% CI: 1.11, 1.25; *p* value < 0.01); level of studies (aOR: 0.72; 95% CI: 0.55, 0.94; *p* value = 0.02) and occurrence of a life-changing crises (aOR: 2.13; 95% CI: 1.28, 3.55, *p* value < 0.01).

**Conclusion:**

The prevalence of depression amongst nursing students in Cameroon is high. Determinants proposed in this study require further assessment to facilitate early identification and management of depression in this high-risk group, in order to limit the negative effects linked with the condition.

## Background

Depression is a common mental disorder worldwide affecting about 350 million people annually [1, 2]. It is a major cause of disability globally [2, 3], as individuals affected with this disorder present with debilitating symptoms which may lead to self-harm or suicide [1].

Nursing students find themselves constantly shuffling their schedule due to clinical practice rotations, night shifts and heavy workload during their studies. They are therefore exposed to great levels of prolonged stress. As a result, nursing students are highly susceptible to depression, with some studies showing that up to 38.7% of individuals in this population are depressed [4].

Depression has a considerable negative impact on the academic performance of nursing students. Depressed students have been shown to have greater absenteeism from lectures and lower cumulative grade point averages (GPA) than non-depressed students [5]. The negative impact of depression on the academic performance of students is even more evident from the considerable variation in the academic performance of students with mild, moderate and severe depression; with higher levels of depression being associated with poorer academic performances [5, 6]. Early identification of depressed students could prove key in limiting the negative consequences of depression, emphasizing the need for mental health facilities within nursing institutions. These facilities should be available for regular evaluation of students and the prompt management of affected ones. In our setting however, there are inadequate mental health assistance services for health professionals and nursing students.

In a previous analysis within this same population of nursing students, we identified that the predictors of burnout syndrome were satisfaction with results and regret of choice of studies [7]. Considering that burnout and depression are different constructs, we set out to determine if burnout is a correlate of depression and to establish other determinants of depression in this high-risk group.

## Methods

### Design

This was a cross-sectional analysis of nursing students recruited conveniently, from a combination of state-owned and private medical institutions in the English-speaking regions of Cameroon between January – April 2018. Consenting participants were recruited using a printed, structured questionnaire, which they were given to answer and return at their convenience. Details regarding the sampling method, sample size, the structuring of the questionnaire and the methods used have been previously described [7].

The study instrument constituted of sections collecting sociodemographic information; and two standard questionnaires – the Patient Health Questionnaire – 9 which was used to assess depression [8] and the Oldenburg burnout inventory (OLBI) which was used to assess burnout [9]. A total of 447 questionnaires were distributed and returned.

### Data management and statistical analysis

Data from returned questionnaires was entered into EPI Info version 7 (CDC, Atlanta) and later exported to STATA version 12 for statistical analysis.

Results were presented as counts (percentages) and means [standard deviation (SD)].

A diagnosis of depression was made if a student had a total PHQ-9 score greater than 4. Depression severity was classified as follows: mild: 5–9; moderate: 10–14; moderately severe: 15–19; and severe: 20–27 [8]. The prevalence of depression amongst these students were calculated in these categories.

Both subscales of the Oldenburg burnout inventory (OLBI) were added to obtain a total score for burnout syndrome after accounting for negatively phrased questions [9]. The mean total OLBI score was calculated in both categories of students (those with or without depression) and compared using a student t-test to assess if burnout was significantly associated with depression.

A univariable analysis comparing sociodemographic and clinical variables as correlates amongst categories of the outcome – depression, was done using the chi-squared test. Significant variables were assessed in a multivariable logistic regression model to determine independent correlates for depression. Variables were purposefully selected into the model at a significance level of 0.1 [10].

## Results

### Sociodemographic characteristics

A total of 447 questionnaires were handed out and returned with 99 (22.45%) from the University of Bamenda, 91(20.63%) from the University of Buea and 251(56.92%) from other private nursing schools (Saint Veronica higher institute of biomedical and nursing sciences Buea and Saint Louis higher institute of health Bamenda). The participants were divided into four levels of studies: Year one (27.75%), Year two (39.73%), Year three (20.54%) and Year four (11.96%).

Most of the participants (81.17%) were female (Table 1). The mean age of the participants was 22.28 ± 3.61 years with an average cumulative grade point average (GPA) of 2.79 ± 0.59 out of a total of 4 (Table 2).

**Table 1:** Univariable analysis for potential categorical correlates of depression among 447 nursing students in the English-speaking regions of Cameroon assessed for depression from January – April 2018

Variable	Category	Total sample		Depression		OR	95% CI	***p*** value
		N	%	n	%			
Gender (*n* = 446)	Male	84	18.83	61	72.62	0.83	0.47, 1.44	0.49
	Female	362	81.17	249	68.78	1.00		
Marital status (n = 446)	Single	401	89.91	286	71.32	0.50	0.26, 0.99	0.03
	Married	45	10.09	25	22.25	1.00		
Personal relationship (*n* = 440) ^a^	Yes	221	50.23	166	75.11	1.60	1.04, 2.48	0.02
	No	219	49.77	143	65.30	1.00		
Difficulties in personal relationship (*n* = 427)	Yes	77	18.03	60	77.92	1.53	0.83, 2.94	0.15
	No	350	81.97	244	69.71	1.00		
Monthly income sufficient (*n* = 407)	Yes	98	24.08	60	61.22	0.63	0.38, 1.05	0.06
	No	309	75.92	221	71.52	1.00		
Course repeated (*n* = 439)	Yes	168	38.27	118	70.24	1.04	0.67, 1.62	0.85
	No	271	61.73	188	69.37	1.00		
Exam re-sited (*n* = 429)	Yes	220	51.28	153	69.55	0.99	0.64, 1.52	0.94
	No	209	48.72	146	69.86	1.00		
Satisfaction with results (*n* = 396)	Yes	122	30.81	85	69.67	0.96	0.59, 1.58	0.88
	No	274	69.19	193	70.44	1.00		
Regret choice of nursing studies (n = 439)	Yes	50	11.39	34	68.00	0.91	0.47, 1.85	0.78
	No	389	88.61	272	69.92	1.00		
Life-changing crises (*n* = 445) ^b^	Yes	19	50.92	17	89.47	1.64	1.06, 2.53	0.06
	No	426	49.08	293	68.78	1.00		
Presence of chronic illness (*n* = 411)^c^	Yes	19	4.27	17	89.47	3.86	0.89, 34.83	0.03
	No	392	95.73	256	65.31	1.00		
Alcohol consumption (n = 446)	Yes	127	28.48	94	74.02	1.34	0.83, 2.20	0.21
	No	319	71.52	217	68.03	1.00		
Recreational drug use (*n* = 444) ^d^	Yes	5	1.13	4	80.00	1.76	0.17, 87.17	0.61
	No	439	98.87	305	69.48	1.00		

^a^ Personal relationship was defined as close connections between two people formed by emotional and sexual interactions; ^b^ Life-changing crises defined as loss of a loved one, physical or sexual trauma and condition of emotional or social instability ^c^ Chronic illnesses included: Asthma, chronic pelvic pain, diabetes mellitus, gastroesophageal reflux disease, chronic peptic ulcer disease, migraines, cerebral lesions and paralysis; ^d^ recreational drugs included: marijuana and tramadol

**Table 2:** Univariable analysis for potential continuous correlates of depression among 447 nursing students in the English-speaking regions of Cameroon assessed for depression from January – April 2018

Variable	N	Total sample				Depression			No depression			*p* value
		Mean	SD	Min	Max	n	Mean	SD	N	Mean	SD	
Age	400	22.28	3.61	17	50	277	21.99	2.99	123	22.92	4.66	0.02
Monthly income in USD	287	32.85	23.46	0	187.68	198	32.14	24.16	89	34.43	29.83	0.44
Number of children	432	0.20	0.68	0	6	302	0.15	0.54	130	0.32	0.93	0.02
Cumulative GPA	306	2.79	0.59	0	4	205	2.76	0.63	101	2.86	0.50	0.20
Level of studies	443	1.17	0.97	0	3	307	1.08	0.96	136	1.36	0.97	< 0.01
Total OBLI score	447	38.04	4.78	22	53	311	38.97	4.83	136	35.91	3.92	< 0.01
Number of hours spent studying	425	4.74	3.10	0	19	292	4.76	3.08	133	4.71	3.15	0.88

*USD* United states dollars, *GPA* cumulative grade point average, *OLBI* Oldenburg burnout inventory; Quantity of alcohol measured in units of alcohol consumed a week

### Prevalence of depression

The overall prevalence of depression in these students (PHQ-9 > 4) was 69.57% (Table 3) and 26.40% of the students had a major depressive disorder (PHQ-9 ≥ 10). For participants with a score of at least one on any of the PHQ-9 questions, 215 (61.60%), 34 (9.74%) and 11 (3.15%) admitted that managing these problems were “somewhat difficult”, “very difficult” and “extremely difficult” respectively.

**Table 3:** Categorisation of prevalence of depression among 447 nursing students in the English-speaking regions of Cameroon assessed for depression from January – April 2018

Category of depression	N	%
Mild depression	193	43.18
Moderate depression	95	21.25
Moderately severe depression	19	4.25
Severe depression	4	0.89
Overall depression	311	69.57

### Determinants of depression

The following variables were significantly associated with depression on univariable analysis: presence of a personal relationship [OR (Odds ratio): 1.60; 95% CI (confidence intervals): 1.04, 2.48, *p* value = 0.02]; marital status (OR: 0.50; 95% CI: 0.26, 0.99; *p* value = 0.03) and presence of a chronic illness (OR: 3.86; 95% CI: 0.89, 34.83, *p* value = 0.03) (Table 1). Depression was also associated with age (p value = 0.02), number of children (*p* value = 0.02), total OLBI score (*p* value < 0.01) (Additional File 1) and level of studies (*p* value < 0.01) (Table 2).

After multivariable logistic regression analysis, independent correlates of depression were found to be: total OLBI score (aOR: 1.18; 95% CI:1.11, 1.25; *p* value < 0.01); level of studies (aOR: 0.72; 95% CI: 0.55, 0.94; *p* value = 0.02) and occurrence of a recent life-changing crises (aOR: 2.13; 95% CI: 1.28, 3.55, *p* value < 0.01) (Table 4).

**Table 4:** Multivariable logistic regression analysis for independent correlates of depression among 447 nursing students in the English-speaking regions of Cameroon assessed for depression from January – April 2018

Correlates	Adjusted Odds Ratios	95% Confidence intervals	***p*** value
**Marital status (Married/Single)**	0.95	0.35, 2.60	0.92
**Personal relationship (Yes/No)**	1.64	0.97, 2.75	0.06
**Sufficient monthly income (Yes/No)**	0.64	0.36, 1.15	0.14
**Life-changing crises (Yes/No)**	2.13	1.28, 3.55	< 0.01
**Presence of chronic illness (Yes/No)**	1.68	0.33, 8.46	0.533
**Age**	0.95	0.87, 1.04	0.28
**Number of children**	0.96	0.56, 1.65	0.89
**Level of studies**	0.72	0.55, 0.94	0.02
**Total OLBI score**	1.18	1.11, 1.25	< 0.01

*OLBI* Oldenburg burnout inventory

## Discussion

In this study, we found out that the prevalence of depression amongst nursing students in the two English-speaking regions in Cameroon was 69.57 and 26.40% of the students had a major depressive disorder. Independent correlates of depression were burnout syndrome, being in the lower years of nursing studies and having a life-changing crises in the previous six months.

The prevalence of depression (69.57%) in nursing students obtained in our study is higher than that obtained in Brazil (19.2%), Iran (38.7%) and China (22.9%) [4, 11, 12]. Indeed, this prevalence was higher than the pooled prevalence (34.0%) of depression in nursing students obtained from a systematic review by Tung et al in 2018; which had Asia as the region with the highest prevalence of depression in nursing students (43.0%) [13]. These differences may be explained by the different tools used to assess depression amongst these students. However, the prevalence obtained from this study was quite similar to the prevalence of depression amongst medical students in Cameroon (65.2%) [14]. This highlights the high prevalence of this condition in health training facilities across the country. Seeking professional help is not a common option adopted by stressed or depressed nursing students [15]. In our setting, students who actually recognise the need for professional assessment and help have no one to run to, given that despite the high prevalence of depression among nursing/medical students recorded in Cameroon, very few of the health training facilities in the country have a dedicated mental healthcare facility or professional. As a result, some of this students develop personalized coping strategies, some of which (smoking, drinking, recreational drug use) may be unhealthy [15, 16]. In Cameroon where there are limited mental health facilities, there is the need for investigation and institution of adequate preventive policies for this special population.

Burnout syndrome was found to be a correlate of depression with depressed students scoring on average one point above non-depressed students on the OLBI score after adjusting for confounders. It is more regularly advanced nowadays that burnout should be regarded as a separate construct from depression [17]. The strongest evidence yet to suggest burnout syndrome as a cause for depression was provided by Hakanen et al from a seven-year prospective study of Finnish dentists, which established a unidirectional relationship showing that burnout was predictive of depressive symptoms and not the other way round [18]. Nonetheless, due to the cross-sectional nature of this study, it is not possible to ascertain the direction of this relationship amongst the nursing students in our study. There is the need for longitudinal studies amongst these students to properly assess temporality.

In the previous published analysis assessing burnout in this group of students, we determined that satisfaction with results was an independent predictor of burnout [7]. Depressed students have been shown to have poorer academic performances [5, 6], and will consequently be less satisfied with their results. This could in turn precipitate burnout, which according to the results herein, lead to depression. The student is therefore trapped in a vicious cycle, detrimental to their health, academic performance and self-confidence.

It was also shown that being in the lower years of nursing studies predisposed participants to depression as the odds of being depressed decreased by 28% as one moved from the lower to the higher years of nursing studies. Similar trends were observed by Sidana et al in 2012 in a medical training facility in India where students in the first year had a higher prevalence of depression than those in the second year, and elsewhere in Sweden [19, 20]. In Cameroon, the highly competitive nature of nursing entrance exams, the harsh realities of the early basic science years in nursing studies and the new experience of living an autonomous life in the early years of university could be overwhelming for some students. In addition, the nature of the nursing curriculum implies students have several hours of lectures weekly which is exhausting both physically and mentally, and equally reduces the amount of time available to engage in social and recreational activities or hobbies which have been shown to have positive effects on mental health and psychological wellbeing [21, 22]. All these could stimulate regrets with respect to choosing the nursing field and consequently culminate in burnout [7], which in turn could lead to depression as demonstrated by this analysis.

Individuals who had a recent life-changing crisis were two times more likely to be depressed. Other studies have also reported a significant association between depression and major life changes [14] and increased suicidal risk among nursing students with depression [23]. Evidence exists to show that recent life-changing crises could lead to recurrence of depressive episodes and mediates the progress of the disease [24]. There are only two state-owned universities offering nursing degrees in the English-speaking regions of the country. Entrance exams into these institutions are highly competitive and students who do not make these exams are left to scramble for the few spots offered by the private sector, at much higher tuition rates. Nursing training in Cameroon is particularly difficult, with a curriculum which is not only stressful but time consuming. Having to handle stressful and time-consuming studies in addition to the extra burden which a major life-changing event is, could prove particularly difficult for some if not most nursing students in Cameroon. This double dose of burden could not only facilitate exhaustion and hence burnout, but could also easily trigger the onset and worsening of depression.

Mental health facilities are generally lacking in Cameroon and health professionals are generally ill-equipped to deal with depression [25]. More so, special populations at a higher risk of depression like trainees in health-related fields and healthcare professionals do not have access to mental health services. There is also a dearth of data on this topic limiting the ability of policymakers to make informed decisions to remedy the situation although a previous study reports similar findings in selected groups (medical students) in Cameroon [14]. There is the need for more longitudinal studies to identify predictors for depression and provide data to help inform policymakers on the need for early detection and initiation of preventive measures to limit its associated morbidity.

### Limitations

This study had the following limitations: the cross-sectional design of the study limits the ability to infer temporal associations and causality between predictors like burnout and depression. Prospective studies are therefore recommended to properly assess this relationship.

## Conclusions

The prevalence of depression amongst nursing students is high. Determinants proposed in this study need to be assessed amongst these students for early identification and management. It is crucial to enhance coping strategies, and also include counselling and treatment interventions in nursing students with depression or burnout syndrome. Prospective studies are also recommended to properly assess the relationship between burnout and depression to help inform health policy.

## Supplementary information

**Additional file 1.** Scatter plot showing the distribution of depression scores assessed using the PHQ-9 on the y-axis burnout syndrome scores assessed using the OLBI on the x-axis of 447 nursing students in the two English-speaking regions of Cameroon.

## Data Availability

The datasets during and/or analysed during the current study are available from the corresponding author on reasonable request.

## Abbreviations

OLBI: OLdenburg burnout inventory
PHQ-9: Patient Health Questionnaire
GPA: Grade point average
MeHe-LTC: Mental health in Liberia, Tanzania and Cameroon
SD: Standard deviation
CI: Confidence interval
OR: Odds ratio
aOR: Adjusted odds ratio
WHO: World Health Organisation
