# Supplementary figures and images for: Determinants of depression among nursing students in Cameroon: a cross-sectional analysis
Source: BMC Nurs. 2020 Apr 17;19:26. doi: 10.1186/s12912-020-00424-y (PMC7165407; doi:10.1186/s12912-020-00424-y)

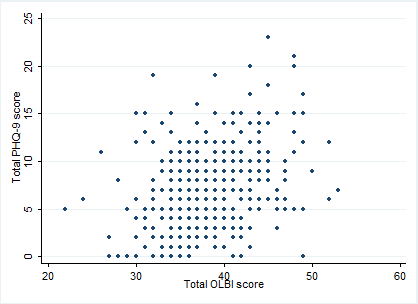

Supplement: Supplementary file 1 — Additional file 1. Scatter plot showing the distribution of depression scores assessed using the PHQ-9 on the y-axis burnout syndrome scores assessed using the OLBI on the x-axis of 447 nursing students in the two English-speaking regions of Cameroon. [file 12912_2020_424_MOESM1_ESM.tif]
